# Matrix Stiffness Affects Endocytic Uptake of MK2-Inhibitor Peptides

**DOI:** 10.1371/journal.pone.0084821

**Published:** 2014-01-06

**Authors:** Jamie L. Brugnano, Alyssa Panitch

**Affiliations:** Weldon School of Biomedical Engineering, Purdue University, West Lafayette, Indiana, United States of America; University of California, San Diego, United States of America

## Abstract

In this study, the role of substrate stiffness on the endocytic uptake of a cell-penetrating peptide was investigated. The cell-penetrating peptide, an inhibitor of mitogen-activated protein kinase activated protein kinase II (MK2), enters a primary mesothelial cell line predominantly through caveolae. Using tissue culture polystyrene and polyacrylamide gels of varying stiffness for cell culture, and flow cytometry quantification and enzyme-linked immunoassays (ELISA) for uptake assays, we showed that the amount of uptake of the peptide is increased on soft substrates. Further, peptide uptake per cell increased at lower cell density. The improved uptake seen on soft substrates *in vitro* better correlates with *in vivo* functional studies where 10–100 µM concentrations of the MK2 inhibitor cell penetrating peptide demonstrated functional activity in several disease models. Additional characterization showed actin polymerization did not affect uptake, while microtubule polymerization had a profound effect on uptake. This work demonstrates that cell culture substrate stiffness can play a role in endocytic uptake, and may be an important consideration to improve correlations between in vitro and in vivo drug efficacy.

## Introduction

Matrix stiffness is an important regulator of cell behavior [1]. Stiffness has been shown to affect cell morphology and spreading [2], [3], proliferation [4], migration [5], apoptosis rate [4], [6], and differentiation [7], [8]. However, most cell studies are performed on tissue culture plastic, which largely fails to replicate the mechanics and microenvironment that cells experience *in vivo*. Tissue culture plastic is commonly cited as having an elastic modulus of approximately 1 GPa, whereas tissues in the body are less than 100 kPa, with brain having an elasticity less than 1 kPa, muscle around 10 kPa, and bone around 100 kPa [7].

The effects of matrix stiffness are typically evaluated by analyzing cell behavior in different gel systems. Stiffness or elasticity can be varied by simply changing the crosslinking density. Several different hydrogel systems have been investigated including polyacrylamide gels [9]–[11], alginate [12], collagen [13], matrigel [14], chitosan [15], and hyaluronic acid [16]. Because substrate stiffness regulates so many cellular functions, we wanted to investigate its role in the uptake of cell-penetrating peptides. Although the exact mechanism of cell-penetrating peptide uptake is still debated, investigators generally agree that uptake occurs via one or more of the endocytic pathways: clathrin-mediated endocytosis, caveolae-mediated endocytosis, and macropinocytosis [17]–[20], or through membrane destabilization or formation of transient pores [21]–[23]. Our lab has designed and reported on a family of peptide inhibitors of mitogen kinase activated protein kinase- activated protein kinase 2 (MAPKAPK2 or MK2), a kinase important in regulating inflammation through the regulation of proinflammatory cytokines [24]–[26]. These inhibitors consist of a cell-penetrating peptide domain for intracellular delivery and a therapeutic domain that inhibits MK2. Recently, we demonstrated that the peptide variant YARAAARQARAKALARQLGVAA (YARA) was taken up primarily through caveolae-mediated endocytosis in mesothelial cells [27]. However, when comparing between data obtained from in vitro cell and in vivo animal models, we observed an unusual effect: concentrations of the YARA MK2 inhibitor peptide required for efficacy in cells ranged from 1000–3000 µM [24], [26]; however, the concentration required for efficacy in animal models was ten to one hundred-fold less, in the range of 10–100 µM [25], [28]–[31]. This phenomenon opposes what is normally observed in the pharmaceutical industry, as drug concentrations must usually increase to demonstrate efficacy when moving from cell culture to animal models due to metabolism and non-uniform distribution within the body. We hypothesized that the discrepancy observed in peptide concentration required to achieve efficacy in studies *in vitro* as compared to studies *in vivo* was due to the unrealistic stiffness of tissue culture polystyrene.

Using a technique pioneered by Pelham and Wang [11] and refined by others [10], the role of substrate stiffness in the uptake of the MK2-inhibitor peptides was investigated. Polyacrylamide gels were chosen as the model substrate for this experiment because stiffness can be modulated by changing the percentage of bisacrylamide crosslinker within the system. Additionally, polyacrylamide gels are clear, non-fluorescent, and have the ability to covalently link proteins to the surface. Unlike most other systems, polyacrylamide gels are inert to protein adsorption and cell adhesion; thus, cellular adhesion can be controlled by functionalizing the gels with an extracellular matrix (ECM) protein. The adhesion of cells to the gel is then solely attributed to cellular binding to the ECM protein.

## Methods

### 2.1 Polyacrylamide Gel Substrate Preparation

Substrates of different stiffness were prepared on 18 mm circle glass coverslips (VWR) following a modified protocol from Tse and Engler [10]. A uniform film of sodium hydroxide (Sigma) was formed on the coverslips by evaporation of 600 µl of 0.1 M sodium hydroxide in a 60°C oven. In the case that uniform coverage was not achieved, 600 µl of water was added to the coverslips and evaporated in a 60°C oven. The coverslips were reacted with 200 µl of (3-aminopropyl) triethoxysilane (Sigma) for five minutes at room temperature under a nitrogen tent, followed by extensive washing with water. The coverslips were then incubated for 30 minutes at room temperature with 0.5% glutaraldehyde (Polysciences). After allowing the coverslips to air dry, polyacrylamide gels were formed on the coverslips under a nitrogen tent. Glass slides (VWR) were covered with 200 µl dichlorodimethylsilane (DCDMS, TCI America) for 5–10 minutes and then washed extensively with water. The polyacrylamide gel stocks were made up from a mixture of millipore water, 2% bis solution (Bio-Rad Laboratories, Inc), and 40% acrylamide solution (Bio-Rad Laboratories, Inc). The “soft” polyacrylamide gel had a final concentration of 0.03% bis solution and 10% acrylamide. The “stiff” polyacrylamide gel had a final concentration of 0.5% bis solution and 10% acrylamide. To these stock solutions, 1/100 volume of 10% ammonium persulfate (APS; Sigma) and 1/1000 volume of N,N,N′,N′-Tetramethylethylenediamine (TEMED; Sigma) was added. A 40 µl volume of the polyacrylamide solution was sandwiched between the coverslip and the DCDMS-treated cover slide. After the polyacrylamide gel polymerized, the coverslips were washed 3 times with water for five minutes. To each coverslip, 200 µl of a 0.8 mg/ml solution of sulfosuccinimidyl-6-[4′-azido-2′-nitrophenylamino]hexanoate (Sulfo-SANPAH, Thermo-Scientific) in millipore water was added. The coverslips were exposed to a 365-nm UV light for 30 minutes to covalently attach the sulfo-SANPAH to the polyacrylamide gels. After washing three times for five minutes each with 50 mM HEPES buffer (Mediatech Inc), pH 8.5, the coverslips were incubated with 0.14 mg/ml fibronectin (BD Biosciences) overnight at 4°C. After three washes with sterile DPBS, the coverslips were sterilized under UV light in a biosafety cabinet and transferred to 12-well plates for cell seeding.

### 2.2 Protein Characterization

To ensure that both soft and hard substrates had equivalent amounts of extracellular matrix attached to their surface, extracellular matrix protein was quantified using a BCA assay Protein Kit (Pierce) with the enhanced test tube protocol according to manufacturer’s instructions. Substrates were made as described, except that they were not sterilized under UV light. Substrates were transferred to sterile 12-well plates (Greiner One), and incubated with 2 ml working reagent (50 parts reagent A to 1 part reagent B) at 60°C for 30 minutes. After cooling to room temperature, the liquid in each well was transferred to a cuvette and absorbance was measured at 562 nm an M5 Spectrophotometer (Molecular Devices) equipped with SoftMax Pro Software (Molecular Devices).

### 2.3 Rheology

The mechanical properties of the polyacrylamide gels were characterized using an AR-G2 rheometer (TA instruments) with a 20 mm standard steel parallel plate geometry. Polyacrylamide gels were made as described and 250 µl of solution was used with a 770 µm gap. A solvent trap was used for all experiments to minimize evaporation. The gelation properties of the polyacrylamide gels were monitored over 45 minutes using an oscillatory stress of 10 Pa and a frequency of 1 Hz. During gelation, the temperature was held constant at 25°C. Because temperature of polymerization has been shown to affect the storage modulus (G′) of polyacrylamide gels [32], the temperature during mechanical characterization closely followed the temperature during gel synthesis. Once gelation was complete, the viscoelastic properties of the gel were tested at 37°C to better simulate the environment that cells experience. Frequency and stress sweeps were performed to determine the linear viscoelastic range of the system. Frequency sweeps occurred at 37°C following a ten minute equilibration. Using an oscillatory stress of 10 Pa, frequency was varied from 0.01 to 100 Hz, measuring 10 points per decade. Stress sweeps occurred at 37°C. Using a frequency of 1 Hz, the oscillatory stress was varied between 0.01 to 100 Pa measuring 10 points per decade. The results obtained were plotted in Origin. Each data point is averaged across 3 independently prepared samples.

### 2.4 Peptide Synthesis and Purification

The MK2-inhibitor peptide YARA, YARAAARQARAKALARQLGVAA, was synthesized on Knorr-amide resin (Synbiosci Corp.) using standard FMOC chemistry. Two different chemistries were used to couple each amino acid. The first coupling reagents were N-hydroxybenzotriazole (HoBt)/N, N′-diisopropylcarbodiimide (DIC) and the second coupling reagents were 2-(1Hbenzotriazole-1-yl)-1,1,3,3-tetramethyluronium hexafluorophosphate (HBTU) and lutidine. For FITC labeled YARA, an aminohexanoic acid spacer was added to N-terminus to serve as a spacer for the addition of FITC isomer 1 (Molecular Probes). The FITC isomer was solubilized in 12∶7∶5 pyridine/DMF/DCM and incubated with the deprotected peptide overnight. A ninhydrin test was used to check complete coupling of FITC to the peptide. Following synthesis, the peptide was cleaved from the resin with a trifluoroacetic acid-based cocktail, precipitated in ether, and recovered by centrifugation. The recovered peptide was dried in vacuo, resuspended in MilliQ purified water, and purified using an FPLC (ÄKTA Explorer, GE Healthcare) equipped with a 22/250 C18 prep-scale column (Grace Davidson). An acetonitrile gradient with a constant concentration of 0.1% trifluoroacetic acid was used to achieve purification. Desired molecular weight was confirmed by time-of-flight MALDI mass spectrometry using a 4800 Plus MALDI TOF/TOF™ Analyzer (Applied Biosystems).

### 2.5 Cell Culture

Human pleural mesothelial cells were obtained from ATCC (CRL-9444). Cells were maintained at 37°C with 5% CO2 and were used between passages 3 and 12. Three different media formulations were used on the mesothelial cells. Two of the three media formulations were complete media formulations and differed only in their base media. Cells were passaged and grown in Media 199 with Earle’s basic salt solution and 0.75 mM L-glutamine (Mediatech, Inc.) supplemented with 1.25 g/L sodium bicarbonate (Sigma), 3.3 nM epidermal growth factor (EGF; MBL International), 20 mM HEPES (Sigma), trace elements mixture B (Mediatech, Inc.), 10% fetal bovine serum (FBS; Mediatech, Inc.), and 1% penicillin/streptomycin (Mediatech, Inc.). Cells were seeded for the 10-plex ELISA experiment in Media 199 without phenol red (Gibco) with the same supplements as mentioned above. The third media formulation was a serum free media consisting of Media 199 without phenol red supplemented with 20 mM HEPES, trace elements mixture B, and 1% penicillin/streptomycin.

For all experiments, mesothelial cells were seeded at either 80,000 cells/well (21,000 cells/cm^2^) or 300,000 cells/well (71,000 cells/cm^2^) in 12-well tissue culture plates containing the polyacrylamide gel substrates or nothing (control). Cells were allowed to adhere and grow overnight. The following day, the substrates were transferred to new 12-well plates to ensure that the response from only those cells grown on the polyacrylamide substrates would be measured and the media was changed to the serum-free media formulation. The following day, cells were treated with a final concentration of 1 ng/ml IL-β (positive control), 1 ng/ml IL-1β+YARA peptide (various concentrations –see figures), or PBS (negative control). After 24 hours, media was collected for cytokine analysis. The number of living cells was determined using the CellTiter 96 AQueous One Proliferation Assay Reagent (Promega) according to the manufacturer instructions. Briefly, 100 µl of reagent was added directly to 500 µl of cells and media. After one hour of incubation in the cell culture incubator, the absorbance was read at 490 nm with a correction at 650 nm using an M5 Spectrophotometer equipped with Softmax Pro software, and cytokine production was normalized to cell number as described above.

### 2.6 Cytokine Analysis

A 10-spot Human Demonstration kit (Meso Scale Discoveries) was used to analyze TNFα production of mesothelial cells according to manufacturer’s instructions. Briefly, plates were warmed to room temperature and incubated with 25 µl of samples and standards for 2 hours at room temperature with vigorous shaking. The detection antibody was then added to the plate and incubated for 2 hours at room temperature with vigorous shaking. After washing three times with PBS (Mediatech, Inc) +0.05% Tween 20 (Sigma), 2X Read buffer was added to the plate and imaged using a Sector Imager 2400A (Meso Scale Discovery). Data was analyzed using the MSD Discovery Workbench Software.

### 2.7 Uptake Characterization: Flow Cytometry

Flow cytometry was used to characterize the uptake of FITC-YARA. Mesothelial cells were seeded on gel substrates or tissue culture plastic, and cultured as previously described. After treating overnight with serum free media, cells were treated with various concentrations of FITC-YARA (refer to figures) or PBS (untreated control) in serum free media and incubated for 24 hours. To collect cells for flow cytometry, cells were washed with PBS, trypsin treated for 5–10 minutes, neutralized with serum-containing media, collected in a 15-ml conical tube and spun down at 300 × g for seven minutes. Cells were washed with PBS, quenched with trypan blue, then washed four to five times with PBS. Once excess trypan blue was washed away, cells were fixed using 1X cytofix buffer (BD). Samples were stored protected from light at 4°C until samples were run on the Cytomics FC500 MPL flow cytometer (Beckman Coulter) equipped with MXP Cytometry List Mode Data Acquisition and Analysis Software. Data acquisition required 10,000 events. Figures were obtained using Flow Jo software.

To characterize uptake on soft substrates, cells were pretreated with 10 mM methyl-β-cyclodextrin (MβCD) for 1 hour or incubated at 4°C for 30 minutes, then treated with 3 µM YARA for 1 hour. Cells were then collected and assayed by flow cytometry as previously described.

### 2.8 Effects of Cytochalasin D and Nocodazole on YARA Uptake

To elucidate the role of the cytoskeleton in the uptake of FITC-YARA, mesothelial cells were treated with various chemicals known to alter the cytoskeleton. Cytochalasin D (Sigma; 5 µg/ml) was used as an inhibitor of stress fiber formation on tissue culture plastic, lysophosphatidic acid (LPA; Sigma; 1,5,and 25 µg/ml ) was used to induce stress fiber formation on soft substrates and tissue culture plastic, and nocodozole (Calbiochem; 1 and 10 µg/ml) was used to inhibit microtubule polymerization on soft substrates. Cells were seeded and grown on substrates or tissue culture plastic as previously described. Following one hour pretreatment with the various inhibitors, cells were incubated with FITC-YARA for 1 hour then processed for flow cytometry.

### 2.9 Uptake Characterization: Confocal Imaging

For confocal images, cells were treated with FITC-YARA for 1 hour, then washed five times with media before imaging live using an FV1000 confocal microscopy equipped with ASW-10 software (Olympus). For all confocal imaging experiments, controls consisting of live, unlabeled cells were included to ensure cells did not autofluoresce.

### 2.10 Staining for F-actin

For F-actin staining, Mesothelial cells were grown as previously described on polyacrylamide substrates, or on tissue culture polystyrene. Cells were fixed in 4% formalin, washed 3X with PBS, permeabilized with a 10 minute incubation with 0.1% Triton-X, then washed 3X with PBS. Cells were incubated with with Phalloidin Alexafluor 488 (Invitrogen) diluted 1∶25 overnight at 4C. Samples were washed 3X with PBS and imaged with a fluorescent microscope.

### 2.11 Statistics

Data is presented as mean ± standard deviation. Statistical analysis was performed with the program Origin. Data was subjected to a single factor ANOVA (α <0.05) and if a significant p-value was found, was processed with a Tukey post-hoc test.

## Results

### 3.1 Rheological Characterization

**Figure 1:**
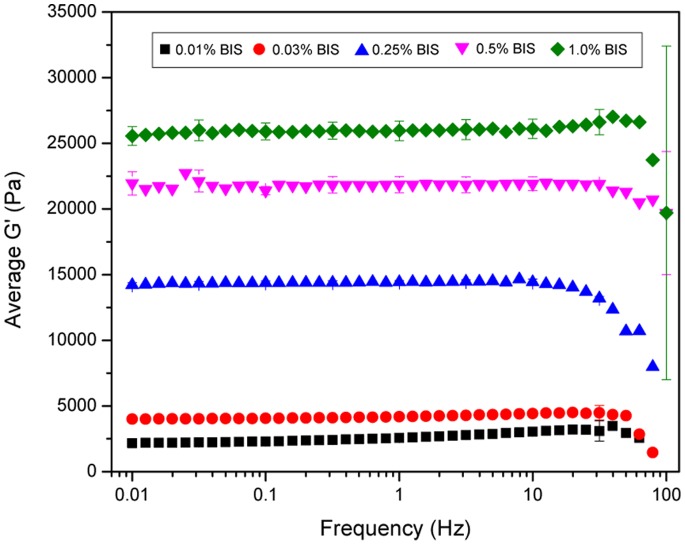
Rheological characterization of polyacrylamide gels that contain 10% acrylamide and various concentrations of bis-acrylamide crosslinker. Data is displayed as mean ± standard deviation.

To determine the optimal polyacrylamide gel system to use for studies, mechanical testing was performed to characterize substrate stiffness with changes in crosslink density (Figure 1). The amount of acrylamide monomer remained at a constant 10% while the bis-acrylamide crosslinker was varied from 0.01% to 1.0%. Substrate stiffness was characterized by measuring the storage modulus G′. By changing the crosslink density of the polyacrylamide gels, the storage modulus was varied from 2.5 kPa to 25 kPa (Figure 1). The choice of which gel system to move forward with was based upon maximizing the difference in mechanical properties between the softest and stiffest gel. However, the stiffest gel with 1.0% bis-acrylamide crosslinker was opaque and would not accommodate light microscopy images of cell attachment. The softest gel with 0.01% bis-acrylamide swelled so much that it prevented the attachment of cells to the extracellular matrix protein grafted to the surface. Thus, the two polyacrylamide gels that were used for further experimentation were the 0.03% bis-acrylamide for the “soft” gel and the 0.5% bis-acrylamide for the “stiff” gel, which equate to a stiffness of approximately 4 and 22 kPa respectively.

### 3.2 Protein Characterization on Polyacrylamide Gels

**Figure 2:**
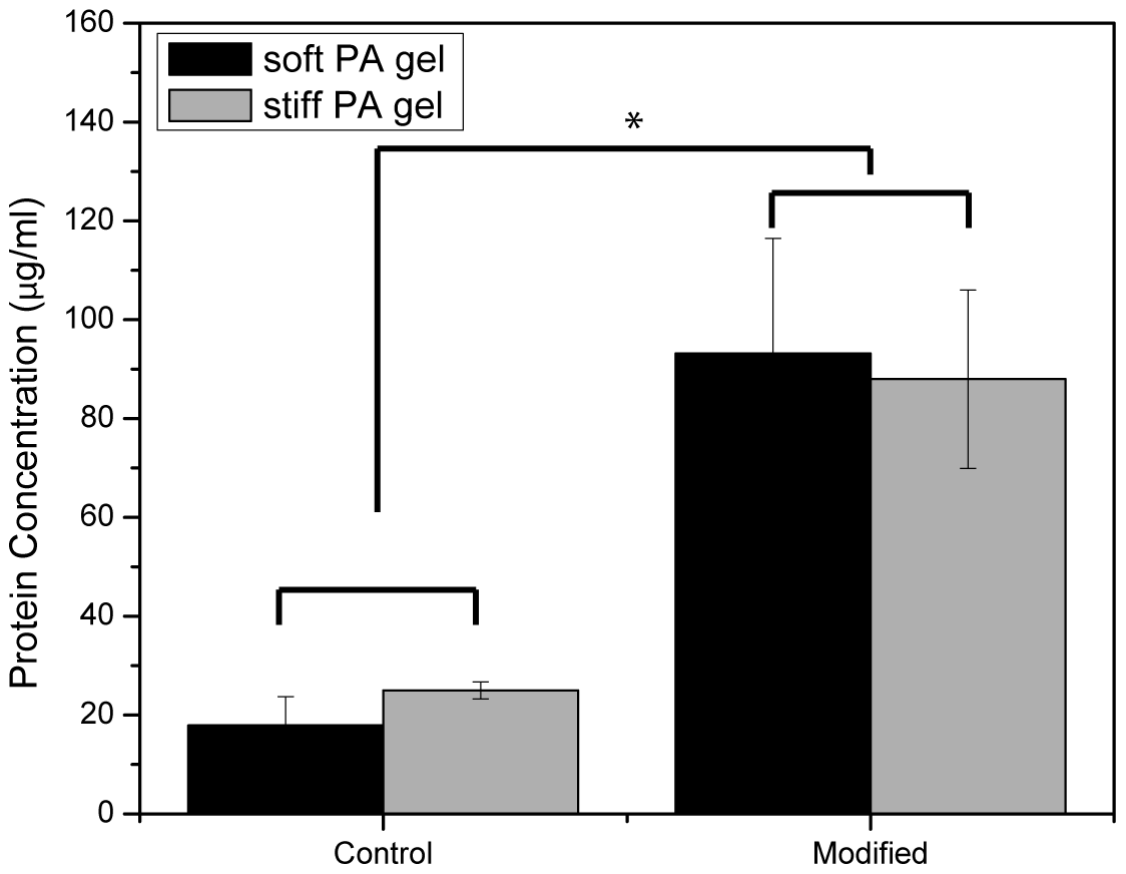
Quantification of fibronectin protein on the surface of polyacrylamide gels. Data is displayed as mean ± standard deviation. Asterisks indicate statistical significance from unmodified control (p<0.05; one-way ANOVA+Tukey post-hoc test).

Fibronectin was covalently grafted to the surface of the polyacrylamide gels to promote cellular adhesion. To determine if there was a difference in the amount of fibronectin present on the gels, a BCA assay was used to quantify total protein concentration on the gels (Figure 2). There was a significant increase in the concentration of protein on the gels grafted with fibronectin (modified) compared to unmodified controls (p<0.05; one-way ANOVA+Tukey post-hoc test), while no difference was found in protein concentration between the modified “soft” and “stiff” gels (p>0.05; one-way ANOVA).

### 3.3 Cellular Morphology on Polyacrylamide Gels

**Figure 3:**
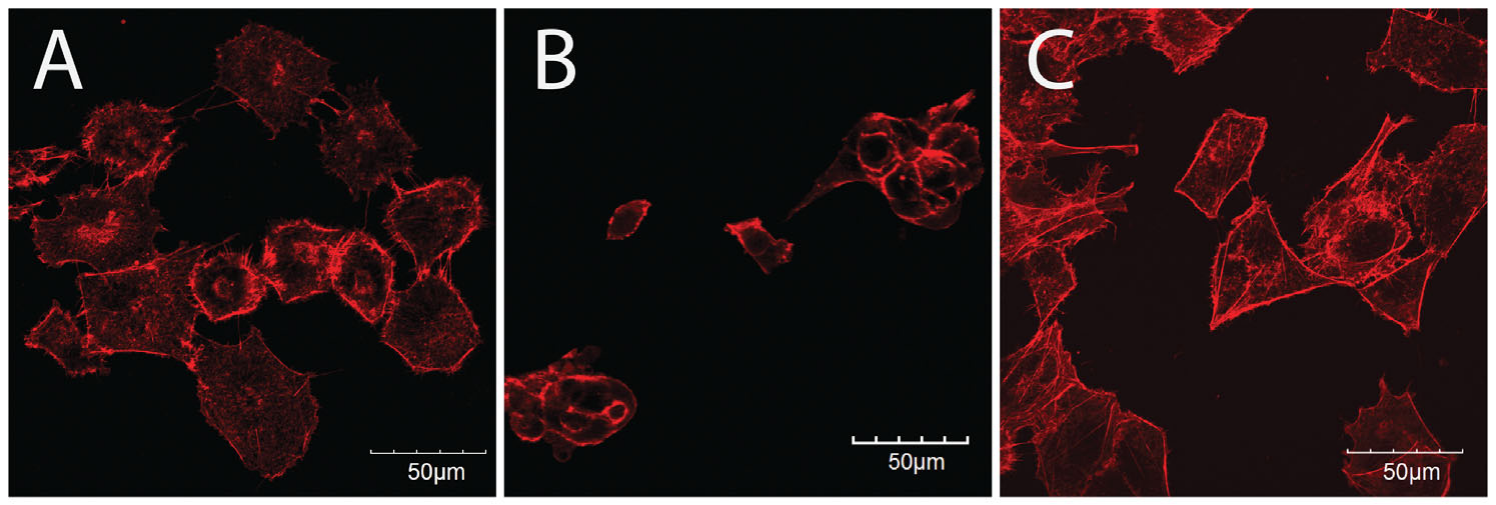
Mesothelial cells stained for F-actin with phalloidin on stiff (A) and soft (B) substrates, and on tissue culture polystyrene (C). Scale bars are 50 µm.

Other investigators have reported a change in cellular morphology in response to matrix stiffness; therefore, we assessed whether mesothelial cells behaved similarly with the stiffnesses examined in this study. Light microscopy images demonstrated a difference in cellular morphology in response to matrix stiffness (data not shown). On tissue culture plastic, mesothelial cells displayed a spread cobblestone appearance. However, on soft substrates, mesothelial cells exhibited a round morphology. Cells on stiff substrates showed an intermediate morphology. This change in cellular morphology was correlated to differences in the actin cytoskeleton (Figure 3). Cells on stiff substrates display prominent F-actin fibers, with small protrusions extending from the periphery of the cells (Figure 3A), similar to what is observed on tissue culture plastic (Figure 3C), while cells on soft substrates are generally smaller (more rounded) and show predominantly cortical actin and actin surrounding the nucleus of the cells (Figure 3B).

### 3.4 Functional Differences on Polyacrylamide Gels

**Figure 4:**
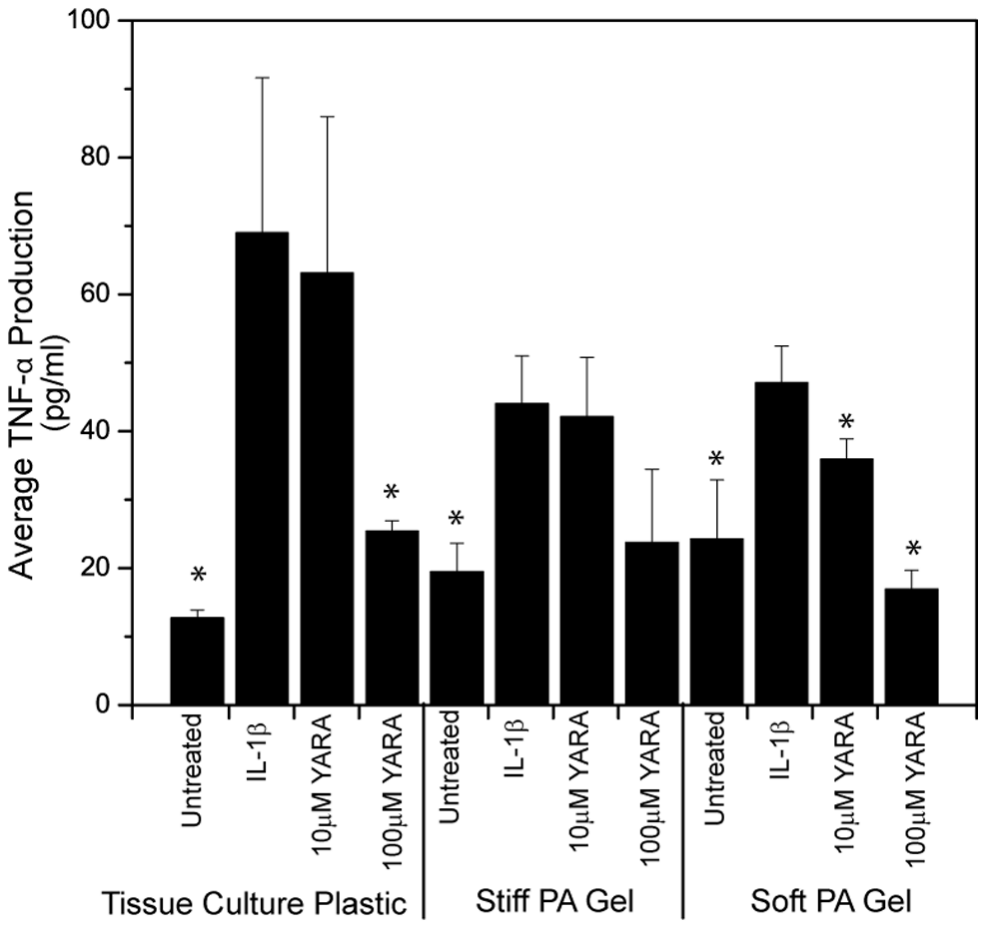
TNF-α cytokine production of mesothelial cells seeded at low density. Cells were treated with the MK2-inhibitor peptide on polyacrylamide gels of different stiffness compared to tissue culture plastic. Cells were seeded at 21,000 cells/cm^2^. Data is presented as mean ± standard deviation. Asterisks indicate statistically significant difference from IL-1β stimulated control within each substrate group (p<0.05; one-way ANOVA+Tukey post-hoc test).

**Figure 5:**
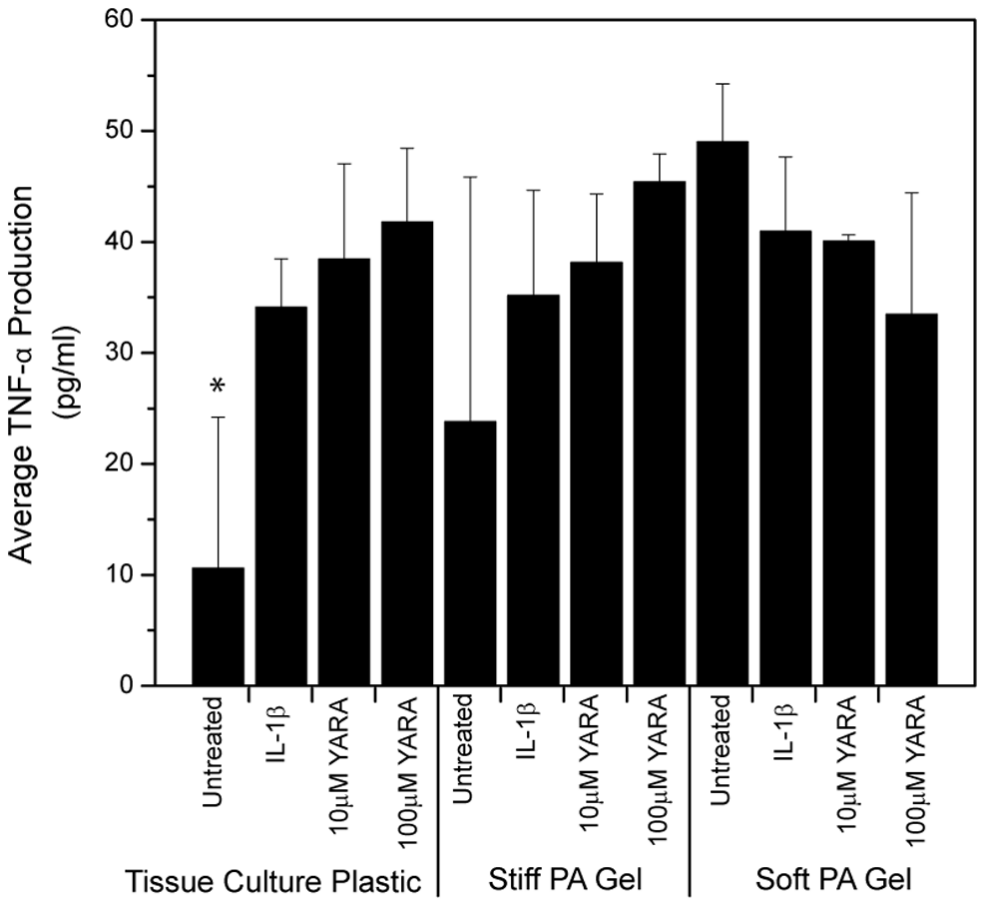
TNF-α cytokine production of mesothelial cells seeded at high density. Cells were treated with the MK2-inhibitor peptide on polyacrylamide gels of different stiffness compared to tissue culture plastic. Cells were seeded at 71,000 cells/cm^2^. Data is presented as mean ± standard deviation. Asterisks indicate statistically significant difference from IL-1β stimulated control within each substrate group (p<0.05; one-way ANOVA+Tukey post-hoc test).

Having demonstrated that mesothelial cells responded morphologically to the change in substrate stiffness, we next characterized whether stiffness affected the uptake and function of the MK2-inhibitor peptide. The MK2-inhibitor peptide was previously shown to regulate proinflammatory cytokine production in mesothelial cells using enzyme-linked immunoassays (ELISA) [26], thus, ELISAs were again used to characterize TNFα secretion in these cells when seeded on the polyacrylamide gels and stimulated with IL-1β and with or without treatment with the MK2-inhibitor peptide YARA (Figures 4 and 5). TNFα production was monitored after 24 hours of treatment. The ELISA results showed increased efficacy of the MK2-inhibitor peptide on soft substrates (Figure 4). On soft substrates, TNF-α production was significantly reduced from the stimulated positive control at a concentration of 10 µM compared to 100 µM on tissue culture plastic (Figure 4; p<0.05; one-way ANOVA+Tukey post-hoc test). In addition, statistical analysis showed that the stiff polyacrylamide substrate is substantially similar to tissue culture polystyrene with respect to TNFα expression. Statistics also show that cells on stiff substrates are less responsive to YARA treatment with respect to suppression of TNFα production. For example, 10 µM YARA treatment on stiff substrates shows a TNFα release level that is statistically higher than untreated stiff and untreated soft substrates, while 10 µM YARA treatment on soft substrates shows a TNFα research that is statistically the same as untreated soft and untreated stiff substrate levels.

These results are dependent upon cell number. When cells were seeded at a high cell density, 71,000 cells/cm^2^, the increased efficacy of the MK2-inhibitor peptide observed on soft substrates disappeared (Figure 5). On tissue culture plastic, cells showed the expected behavior with an increase in cytokine production with IL-1β treatment, however, the concentrations of MK2-inhibitor peptide used did not reverse this effect.

### 3.5 Intracellular Uptake on Polyacrylamide Gels

**Figure 6:**
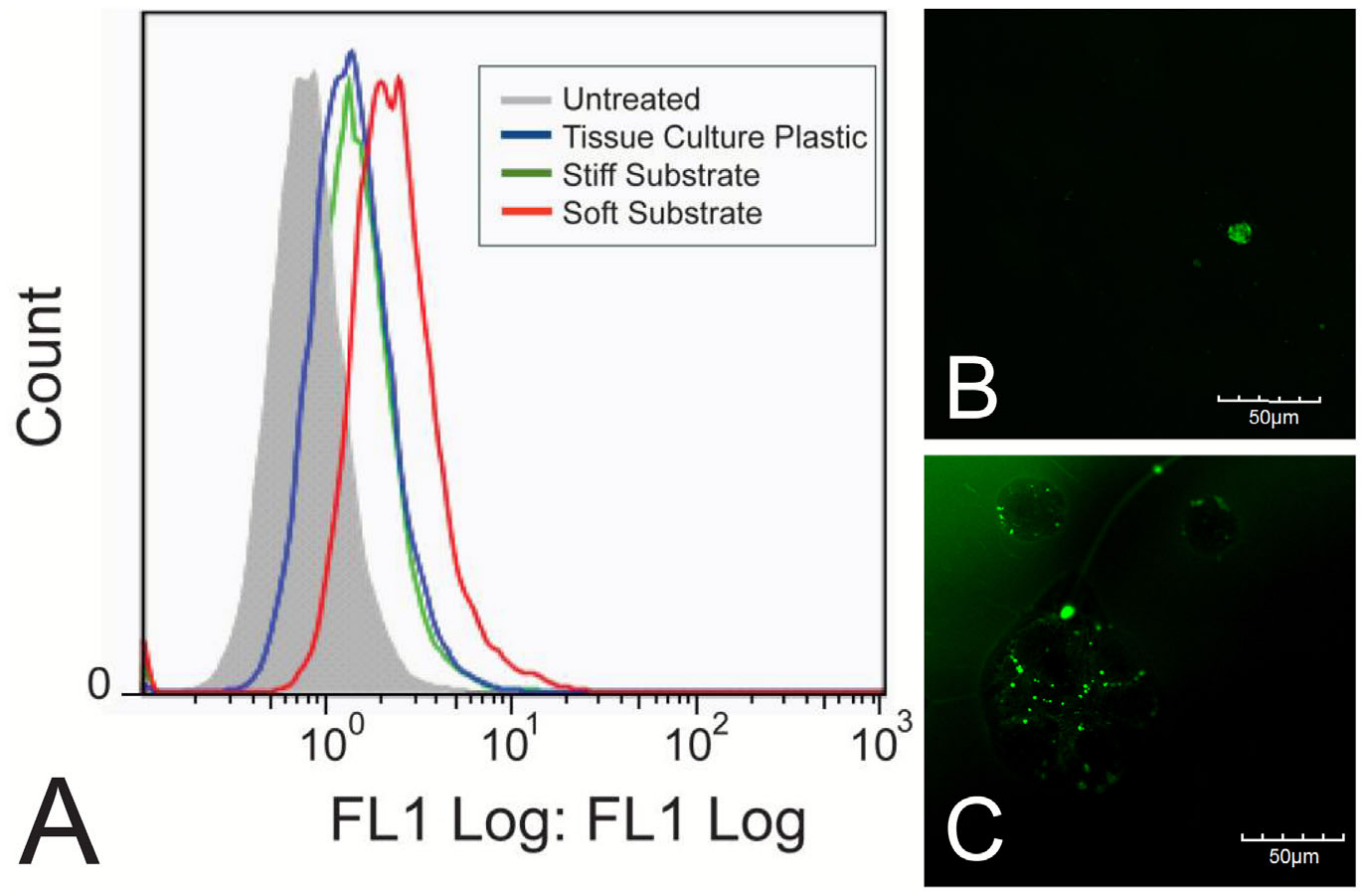
Uptake of 3 µM FITC-YARA in mesothelial cells seeded at low density. Cells were seeded at 21,000 cells/cm^2^ and uptake was evaluated using (A) flow cytometry or (B) confocal microscope on tissue culture plastic, and (C) confocal microscope on polyacrylamide gel substrates. The green fluorescence represents peptide taken up into the cells. Scale bar is 50 µm.

To determine if uptake of the MK2-inhibitor peptide differed as a result of substrate stiffness, the peptide was modified with an N-terminal FITC-label and intracellular uptake was quantified using flow cytometry (Figure 6A), and evaluated qualitatively using fluorescent microscopy (Figure 6B and C). Cells were seeded on tissue culture polystyrene (6B), or substrates (6C) at low density (21,000 cells/cm^2^) to mimic the conditions from the functional results and treated with FITC-YARA for 24 hours. Cells treated on soft substrates show approximately double the uptake of FITC-YARA compared to cells treated on tissue culture plastic, while uptake of FITC-YARA on stiff substrates is similar to that on tissue culture plastic (Figure 6A). The increased uptake of FITC-YARA on soft substrates compared to tissue culture plastic was confirmed visually with confocal imaging, where more peptide (green fluorescence) can be seen in cells on soft substrates (Figure 6C) as compared to cells on tissue culture polystyrene (Figure 6B). All cells, regardless of substrate, treated with FITC-YARA showed increased intracellular uptake compared to untreated controls.

**Figure 7:**
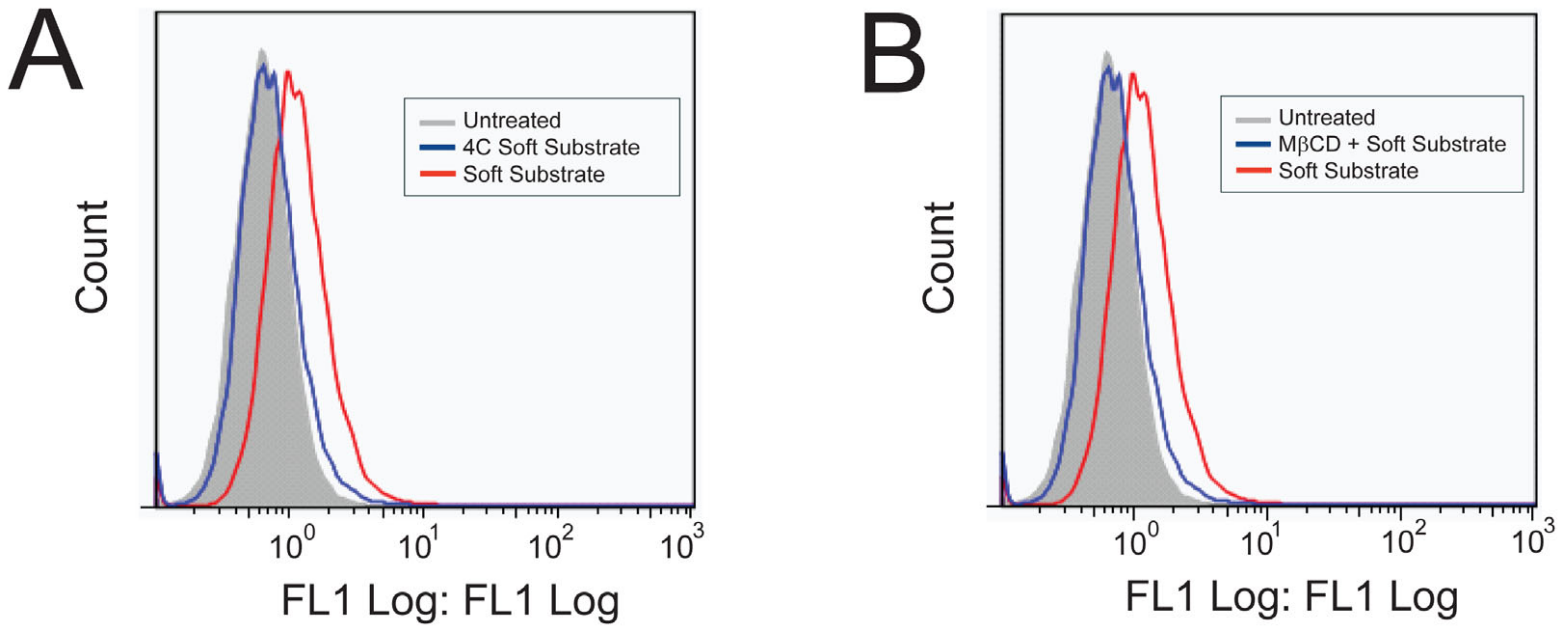
Inhibition of 3 µM FITC-YARA uptake on soft polyacrylamide gel substrates. Uptake is inhibited at 4°C (A) and with the pharmacological inhibitor MβCD (B).

To confirm that uptake occurs through similar mechanisms on the soft polyacrylamide gel compared to tissue culture plastic [27], we characterized the mechanism of uptake by treating the cells with FITC-peptide at 4°C to determine if uptake is energy-dependent (a requirement of endocytic uptake) and with a pharmacological inhibitor of caveolae-mediated endocytosis, methyl-β-cyclodextrin (MβCD). Figure 7A and B shows that uptake of FITC-YARA on soft substrates is inhibited at low temperatures and is inhibited with MβCD pretreatment, similar to what has been observed on tissue culture plastic [27].

**Figure 8:**
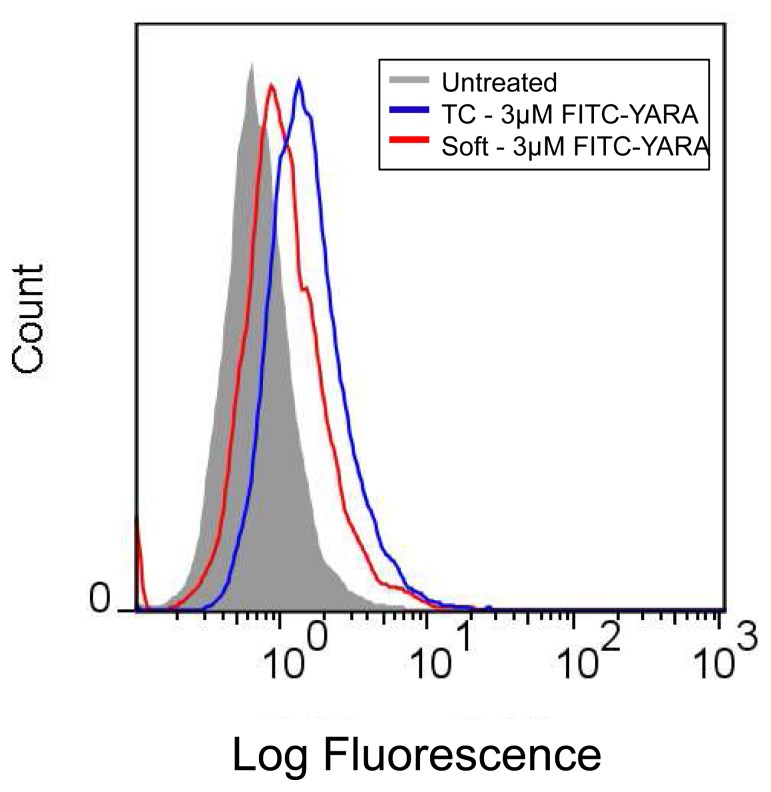
Uptake of 3 µM FITC-YARA in mesothelial cells seeded at high density. Cells on polyacrylamide gel substrates or tissue culture plastic were seeded at 71,000 cells/cm^2^ and uptake was evaluated using flow cytometry.

In addition, we were interested in evaluating uptake as a function of initial seeding density. As shown in Figure 8, seeding density does change the uptake of FITC-YARA. A high initial cell seeding density decreased the amount of FITC-YARA that was endocytosed by mesothelial cells seeded on soft substrates compared to tissue culture plastic. This was completely opposite of what was observed at a lower cell density (Figure 6A) and agreed with functional results: increased cell density decreased uptake of peptide and efficacy of cytokine suppression (Figures 4 and 5).

**Figure 9:**
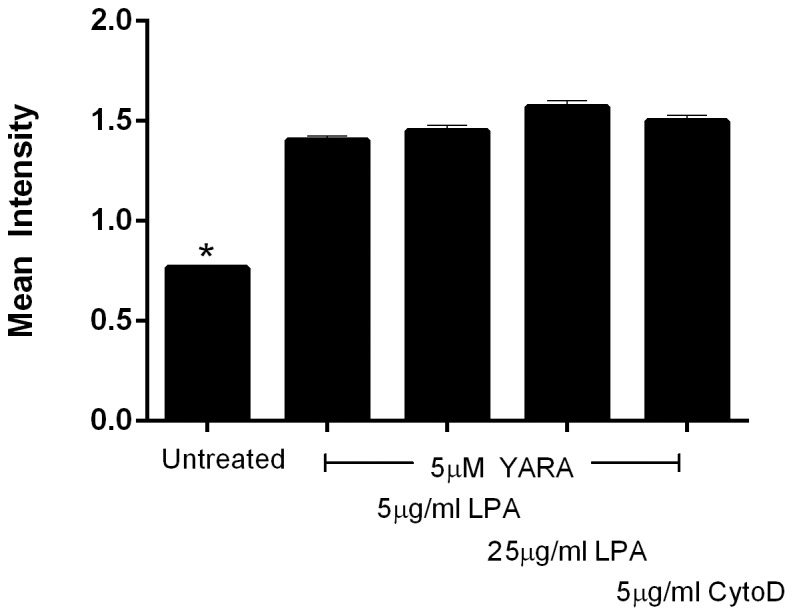
Effect of actin polymerization on uptake of 3 µM FITC-YARA. Cells were seeded on soft substrates at 21,000 cells/cm^2^ and treated with YARA and 5 or 25 µg/ml LPA, or seeded on TCPS and treated with YARA and 5 µg/ml CytoD. Uptake was evaluated using flow cytometry.

Because cells cultured on tissue culture polystyrene showed more pronounced actin stress fibers than those grown on polyacrylamide substrates, we evaluated the effect of actin filaments on YARA uptake using flow cytometry. Using LPA, we induced actin filament formation and using cytochalasin D we disrupted actin filament formation. While cells treated with peptide showed an increased fluorescent signal, indicative of peptide uptake, as compared to untreated cells, treatment with LPA, or treatment with cytochalasin D, had no effect on peptide uptake (Figure 9). This data suggests that peptide uptake is not affected by actin polymerization.

**Figure 10:**
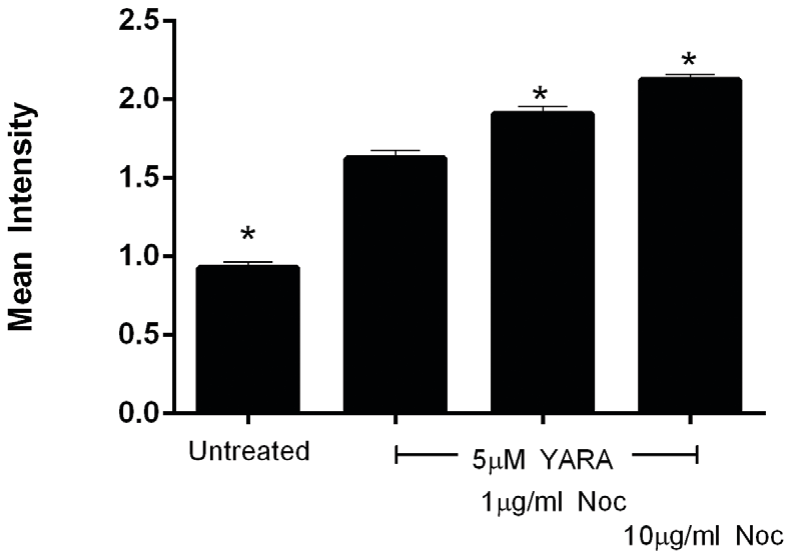
Effect of microtubule polymerization on uptake of 3 µM FITC-YARA. Cells were seeded on soft substrates at 21,000 cells/cm^2^ and treated with YARA and 1 or 10 10 µg/ml of nocodazole. Uptake was evaluated using flow cytometry.

Endosome trafficking is dependent on microtubules, thus, nocodazole was used to interfere with microtubule polymerization to evaluate the effects of microtubules on peptide uptake. Cells treated with nocodazole showed a pronounced increase in YARA uptake, in a dose dependent manner, as compared to untreated cells (Figure 10). This data confirms the importance of microtubules in YARA uptake or trafficking.

## Discussion

In this paper, we provide evidence that matrix stiffness regulates intracellular uptake of MK2-inhibitor peptides. Not only is there an increase in uptake as observed by flow cytometry (Figure 6), but there is a functional difference as characterized by the production of proinflammatory cytokines (Figures 4 and 5). Based on the quantification of surface fibronectin protein, which showed equivalent amounts of ECM protein between the soft and stiff polyacrylamide gel, the difference in YARA uptake can be attributed directly to the effect of stiffness. Characterization of the fibronectin protein is important because several investigators have demonstrated that cells respond morphologically to varying amounts of matrix proteins [33]–[35]. We wanted to ensure that the cell response observed could be appropriately attributed to matrix stiffness and not the matrix proteins.

Importantly, the mechanism of uptake of FITC-YARA does not appear to change when cells are seeded on soft substrates. Mesothelial cells seeded on tissue culture plastic show inhibition of uptake at 4°C and with the removal of cholesterol using the pharmacological inhibitor, MβCD [27]. Figure 7 demonstrated that FITC-YARA uptake on soft substrates was also energy- and cholesterol-dependent.

MK2 is known to regulate the expression of several proinflammatory cytokines, however we focused on the down regulation of TNF-α protein expression as a marker of YARA activity [36]. In previous studies we have noted that cytokine expression is down regulated at 6 hours and continues to be down regulated at 24 hours with the difference between expression in control and treated cells maximized at the later time point [29]. We believe that the prolonged ability of the peptide to suppress cytokine protein expression is due to uptake into and prolonged residence time in caveolae. This is consistent with work by Kim, et al. where they saw prolonged retention of TAT-M13-phage in caveolae [37], and is subject to further investigation.

The concentration required to demonstrate efficacy in this cell line was equivalent to that observed in animal models when cells are seeded at low densities on polyacrylamide substrates. The importance of cell density must not be overlooked. Previous studies in our laboratory indicated that confluent mesothelial cells seeded on tissue culture plastic exhibited a decrease in cytokine production only when treated with very high concentrations of peptide (3000 µM) [26]. However, when cells were seeded at a much lower density on tissue culture plastic, there was evidence of efficacy at 100 µM [27]. Mesothelial cells were initially cultured and evaluated for efficacy at such a high density because it was thought that the cell-cell contact was best for mimicking the *in vivo* environment where the cells form a monolayer called the mesothelium [38]. However, based on the results presented herein, this assumption is clearly incorrect. Cell context is critical to cell behavior, and cells change their genetic profile base on cell-cell contacts, how close to the edge of a culture they are, and even cell density [39]. Similar to other mammalian cells, mesothelial cells have the ability to change phenotype [40], and it is possible that at high seeding concentrations the mesothelial cells are differentiating and are thus not as responsive to YARA. It is also possible that the rate of endocytosis is decreased at high seeding densities, due to phenotypic and metabolic changes at high cell densities. This latter possibility is consistent with the studies of Snijder, et al. where they used both modeling and experiment to show that the rate of endocytosis changed with cell density, and how close the edge of a cell islet the individual cell was [41]. Thus, phenotypic changes due to cell density may explain why such variability is observed in YARA efficacy when cells are seeded at high concentrations (Figure 5), and further explain the high concentration of YARA required to observe efficacy at confluence.

Both substrate stiffness and the extracellular matrix have previously been shown to regulate non-viral gene delivery [4], [42], [43]. In these cases, the gene transfer efficiency of plasmid-DNA increased with increasing density of RGD peptides [42], with fibronectin coating (compared to collagen) [43] and with increasing stiffness [4]. The enhanced efficiency of uptake with higher density of RGD peptides and increased stiffness is attributed to an increase in cellular proliferation, while the enhanced uptake with fibronectin coating is attributed to the promotion of endocytic uptake by clathrin-mediated endocytosis [4], [42]. That stiffness has an opposing role in plasmid-DNA uptake compared to our MK2-inhibitor peptide is not surprising. Plasmid-DNA is often cited as being taken up through clathrin-mediated endocytosis, while the MK2-inhibitor in mesothelial cells gets taken up mainly through caveolae-mediated endocytosis [27]. While proliferation can be important for uptake, as seen in the case of plasmid DNA-uptake [8], it does not appear to have the same effect in the case of YARA uptake; qualitative evaluation in our system confirmed that cells on TCPS proliferated at a higher rate than those on soft substrates, while those on soft substrates took in more YARA. Thus, it is possible that mechanism of endocytic uptake could be a tool used to predict how substrate stiffness will affect uptake.

Although we have shown that matrix stiffness and cell density both affect uptake, we have yet to determine the reason for this observation. We hypothesized that the actin cytoskeleton might be responsible for the differential uptake between tissue culture plastic and polyacrylamide gels. Actin stress fibers are affected by substrate stiffness [2]. Actin stress fibers are also regulated by cell-cell contact [2]. Cells on soft substrates do not typically exhibit stress fibers, however, when cells on soft substrates are in cell-cell contact, stress fibers reappear [2]. The actin cytoskeleton is critical in caveolae-mediated endocytosis, and is necessary for the closure and initial uptake of caveolar vesicles [44]. Other investigators have shown that increased density of organized stress fibers impedes clathrin-mediated endocytosis [45]. Furthermore, stress fibers are not prominent in cells *in vivo* [2]. However, our data suggests that YARA uptake is independent of the state of actin polymerization since neither LPA nor cytochalasin D affected YARA uptake (Figure 9).

Microtubules are also important in endosome trafficking [46]. In this study microtubules were shown to be important in YARA uptake or trafficking since nocodazole treatment significantly enhanced YARA uptake. Microtubules are confirmed to affect endosome trafficking including recycling to the plasma membrane; thus, it is likely that disruption of microtubules does not increase the rate of endocytosis of YARA, but delays recycling of YARA to the membrane. The end result of microtubule disruption and delayed recycling is accumulation of YARA within the cells. Uptake appears to be independent of actin polymerization, while accumulation of YARA within the cell is dependent upon microtubule polymerization.

Understanding how substrate stiffness affects intracellular uptake has broad implications in the design of drug screening platforms, both in screening potential drugs for evidence of efficacy and for understanding how uptake might differ in cells within a diseased state. Several different disease states are characterized by changes in tissue rigidity due to inflammation, fibrosis, calcification, or other biochemical changes within the tissue. Understanding whether a drug is influenced by tissue rigidity can help physicians choose therapies that may be more effective for the patient, depending on the stage of the disease.
